# Cannabinoid receptors contribute to astroglial Ca^2+^-signalling and control of synaptic plasticity in the neocortex

**DOI:** 10.1098/rstb.2014.0077

**Published:** 2014-10-19

**Authors:** Seyed Rasooli-Nejad, Oleg Palygin, Ulyana Lalo, Yuriy Pankratov

**Affiliations:** School of Life Sciences, University of Warwick, Coventry, UK

**Keywords:** glia–neuron interaction, endocannabinoid, LTP, vesicular release, synaptic current, d-serine

## Abstract

Communication between neuronal and glial cells is thought to be very important for many brain functions. Acting via release of gliotransmitters, astrocytes can modulate synaptic strength. The mechanisms underlying ATP release from astrocytes remain uncertain with exocytosis being the most intriguing and debated pathway. We have demonstrated that ATP and d-serine can be released from cortical astrocytes *in situ* by a SNARE-complex-dependent mechanism. Exocytosis of ATP from astrocytes can activate post-synaptic P2X receptors in the adjacent neurons, causing a downregulation of synaptic and extrasynaptic GABA receptors in cortical pyramidal neurons. We showed that release of gliotransmitters is important for the NMDA receptor-dependent synaptic plasticity in the neocortex. Firstly, induction of long-term potentiation (LTP) by five episodes of theta-burst stimulation (TBS) was impaired in the neocortex of dominant-negative (dn)-SNARE mice. The LTP was rescued in the dn-SNARE mice by application of exogenous non-hydrolysable ATP analogues. Secondly, we observed that weak sub-threshold stimulation (two TBS episodes) became able to induce LTP when astrocytes were additionally activated via CB-1 receptors. This facilitation was dependent on activity of ATP receptors and was abolished in the dn-SNARE mice. Our results strongly support the physiological relevance of glial exocytosis for glia–neuron communications and brain function.

## 1. Introduction

Cannabinoid receptors are important modulators of synaptic transmission, implicated in the short- and long-term plasticity of inhibitory and excitatory synapses in many regions of the central nervous system [1]. Endogenous cannabinoid (endocannabinoid, eCB) signalling is involved in a variety of brain functions including memory, cognition, pain perception and movement, and dysregulation of the eCB signalling is related to many neuropsychiatric disorders such as depression and anxiety [1–3]. Thus, endocannabinoids and their receptors have attracted great interest in neurophysiological research, including their potential role as therapeutic targets [3,4].

Endocannabinoids are lipid messengers that are produced via a complex, Ca^2+^-regulated cascade. The two widespread and best characterized eCBs are anandamide (AEA) and 2-arachidonoyl-glycerol. Endocannabinoids can be released by variety of pathways, with the principal pathway believed to be a retrograde release from post-synaptic neurons. This event can be triggered either as a result of calcium elevations in post-synaptic neurons or as a result of activation of post-synaptic metabotropic glutamate receptors (mGluRs) [3,5,6]. Endocannabinoids can activate at least two types of G-protein-coupled receptors, correspondingly named CB1 and CB2 receptors [1,3,6]. In central neurons, CB1 receptors are mainly localized pre-synaptically, both in the excitatory and inhibitory neurons [5–7]. In addition to neurons, eCB receptors have been recently found in astrocytes of several brain regions [8–11].

The most prominent and well-established action of neuronal CB1 receptors is a pre-synaptic decrease of neurotransmitter release, mainly by retrograde signalling [1,2,5,6]. Depending on the type of neuron, this effect can take the form of depolarization-induced suppression of inhibition [6,12] or depolarization-induced suppression of excitation [13]. This kind of short-term synaptic plasticity relies on the release of eCBs from post-synaptic neurons and their diffusion to pre-synaptic CB1 receptors, which then downregulate voltage-gated Ca^2+^-channels [1,6,14].

The CB1 receptors can also take part in long-term synaptic plasticity. Their role in long-term synaptic depression in the neocortex and hippocampus has been widely reported [1,14,15]. Endocannabinoid-dependent long-term depression (LTD) can be induced in the absence of post-synaptic NMDA receptor activity and calcium elevations, but requires activation of post-synaptic class I mGluRs to trigger eCB synthesis via phospholipase C (PLC) activation. The expression of eCB-LTD is related to activation of Gi/o protein coupled to pre-synaptic CB1 receptors, leading to reduction in neurotransmitter release [14,15]. There is also growing evidence that eCBs contribute to regulation of long-term potentiation (LTP) [1,14,16], including recent data obtained in CB1 knockout mice. Still, different studies have reported diverse effects of CB1 receptors. Several earlier studies have shown that pharmacological blockade of CB1 receptors does not have any significant effect on LTP induction [16,17]. However, more recent studies showed enhancement of LTP in CB1 knockout mice *in vivo* [18]. By contrast, there are studies that have shown complete abolishment of LTP in hippocampal slices perfused with CB1 receptor antagonists [19,20]. Reports on the role of eCBs in the modulation of LTP induction in the neocortex are more scarce and controversial. Observation of negative modulation of LTP in prefrontal cortex by a high concentration of the CB1 agonist WIN55,212-2 [21] contrasts with a reported lack of effect of both WIN55,212-2 and CB1 antagonist AM251 (5 μM) on LTP in visual cortex [15]. The reason for these contradictory results could be the presence of CB1 receptors in both excitatory and inhibitory synapses [1,14].

Activation of astrocyte CB1 receptors leading to glial modulation of synaptic function can also add another layer of complexity to eCB signalling. Recent studies highlighted the role of eCBs in glial signalling and glia–neuron interaction [8–11]. In contrast to neurons, CB1 receptors in astrocytes can be coupled to PLC via Gq/11-proteins and thereby can increase the intracellular Ca^2+^ level [10] and, plausibly, trigger an exocytotic release of gliotransmitters, such as glutamate, ATP or d-serine [22]. It has been shown that CB1 receptors of hippocampal astrocytes can trigger release of glutamate, which in turn can activate post-synaptic NMDA receptors on CA1 pyramidal neurons or pre-synaptic mGuR1 receptors [9,10]. The latter mechanism was reported to cause short-term facilitation of transmitter release at some population of excitatory synapses. In neocortex, astrocyte CB1 receptors can mediate spike timing-dependent LTD between pyramidal neurons in layer IV and layer II/III, most likely via triggering release of glutamate and activating pre-synaptic NMDA receptors [11]. Importantly, physiological relevance of astrocytic eCB signalling has been supported by the recent *in vivo* demonstration of Δ9-tetrahydrocannabinol-induced long-lasting suppression of excitatory synaptic transmission in hippocampus; this effect was selectively abolished by glia-specific knockout of CB1 receptor [8]. The mechanism of eCB-dependent depression involved, presumably, release of glutamate from astrocytes leading to activation of post-synaptic NMDA receptors and subsequent endocytosis of AMPA receptors [8]. These results contrast with the observation of eCB-mediated potentiation of synaptic transmission in hippocampus via astrocytic CB1 receptors [10].

Thus, the detailed mechanism by which eCB-activated astrocytic Ca^2+^-signalling affects synaptic function needs further investigation. In particular, it is yet to be verified that astroglial CB1 receptors can activate a vesicular release of gliotransmitters. Also, previous studies of the eCB-mediated component of glia–neuron interaction have been focused on the putative role of glutamate as the gliotransmitter, whereas the repertoire of gliotransmitters is much broader. Astrocytes can affect long-term plasticity by releasing the NMDA receptor co-agonist d-serine [23] and ATP [24]. It has also been demonstrated that astrocytes release ATP by SNARE-dependent exocytosis [24,25] and that astroglia-derived ATP can activate neuronal P2X receptors, which, in turn, downregulate inhibitory synaptic transmission in the neocortex [25]. In this paper, we demonstrate that eCB-mediate Ca^2+^-signalling can activate exocytosis of gliotransmitters, in particular ATP, and this mechanism contributes to modulation of synaptic plasticity in the neocortical neurons. In our present work, we use a combination of techniques, including the ‘sniffer’-cell approach [25], and transgenic mice with inducible expression of the dominant-negative (dn)-SNARE domain selectively in astrocytes [24].

## 2. Material and methods

Experiments were performed on astrocytes and neurons from somatosensory cortex of dn-SNARE transgenic mice [24,26], their wild-type (WT) littermates and transgenic mice expressing enhanced green fluorescent protein (EGFP) under the control of the glial fibrillary acidic protein (GFAP) promoter [27,28]. Data obtained in the experiments on GFAP-EGFP mice did not differ significantly from data obtained in the WT mice. For clarity, all data referred to here as WT are reported solely for WT littermates to dn-SNARE mice; usage of GFAP-EGFP mice was explicitly stated where appropriate.

### (a) Slice and cell preparation

Mice (8–12 weeks) were anaesthetized by halothane and then decapitated, in accordance with UK legislation. Brains were removed rapidly after decapitation and placed into ice-cold physiological saline containing (mM): NaCl 130, KCl 3, CaCl_2_ 0.5, MgCl_2_ 2.5, NaH_2_PO_4_ 1, NaHCO_3_ 25, glucose 15, pH 7.4, gassed with 95% O_2_–5% CO_2_. Transverse slices (280 μm) were cut at 4°C and then placed in physiological saline containing (mM): NaCl 130, KCl 3, CaCl_2_ 2.5, MgCl_2_ 1, NaH_2_PO_4_ 1, NaHCO_3_ 22, glucose 15, pH 7.4, and kept for 1–4 h prior to cell isolation and recording.

Astrocytes and neurons were acutely isolated using the modified ‘vibrating ball’ technique [12,25,27,28]. The glass ball (200 µm diameter) was moved slowly some 10–50 µm above the slice surface, while vibrating at 100 Hz (lateral displacements 20–30 µm). This technique preserves the function of membrane proteins and therefore is devoid of many artefacts of enzymatic cell isolation and culturing procedures. The composition of the external solution for all isolated cell experiments was (mM): 135 NaCl; 2.7 KCl; 2.5 CaCl_2_; 1 MgCl_2_; 10 HEPES, 1 NaH_2_PO_4_, 15 glucose, pH adjusted with NaOH to 7.3. Astrocytes were identified by their morphology under differential interference contrast microscopy, EGFP fluorescence (astrocytes from dn-SNARE and GFAP-EGFP mice) or staining with sulforhodamine 101 (astrocytes from WT mice). After recordings, identification of astrocytes was confirmed by functional properties (high potassium conductance, low input resistance and strong activity of glutamate transporters) as described previously [25,27,28].

### (b) Electrophysiological recordings

Whole-cell voltage-clamp recordings from neocortical neurons and astrocytes were made with patch pipettes (4–5 MΩ for neurons and 6–8 MΩ for astrocytes) filled with intracellular solution (in mM): 110 KCl, 10 NaCl, 10 HEPES, 5 MgATP, 10 EGTA, 1 CaCl_2_, pH 7.35. Currents were monitored using an AxoPatch200B patch-clamp amplifier (Axon Instruments, USA) filtered at 2 kHz and digitized at 4 kHz. Experiments were controlled by PCI-6229 data acquisition board (NI, USA) and winfluor software (Strathclyde University, UK); data were analysed by self-designed software. Liquid junction potentials were compensated with the patch-clamp amplifier. Series and input resistances were, respectively, 5–7 MΩ and 500–1100 MΩ in neurons and 8–12 MΩ and 50–150 MΩ in astrocytes; both series and input resistance varied by less than 20% in the cells accepted for analysis. For activation of synaptic inputs, axons originating from layer IV–VI neurons were stimulated with a bipolar coaxial electrode (WPI, USA) placed in the layer V close to the layer IV border, approximately opposite the site of recording; stimulus duration was 300 µs. The stimulus magnitude was set three to four times higher than minimal stimulus adjusted to activate the single-axon response in the layer II pyramidal neurons as previously described [27–29]. In order to trigger synaptically driven astroglial Ca^2+^-transients, the single episode of theta-burst stimulation (TBS; high-frequency stimulation, HFS) was delivered; an HFS episode consisted of five pulses of 100 Hz stimulation, repeated 10 times with 200 ms intervals (total 50 pulses per episode). For induction of long-term plasticity, two or five such HFS episodes were used.

### (c) Multi-photon fluorescent Ca^2+^-imaging in astrocytes

To monitor the cytoplasmic free Ca^2+^ concentraton ([Ca^2+^]_i_) *in situ*, astrocytes of neocortical slices were loaded by 30 min incubation with 1 µM of Rhod-2AM or Oregon Green BAPTA-2 at 33°C. Two-photon imaging of neurons and astrocytes was performed using a Zeiss LSM-7MP multi-photon microscope coupled to a SpectraPhysics MaiTai pulsing laser; experiments were controlled by ZEN LSM software (Carl Zeiss, Germany). Images were further analysed off-line using ZEN LSM (Carl Zeiss) and ImageJ (NIH) software. The [Ca^2+^]_i_ levels were expressed as Δ*F*/*F* ratio averaged over region of interest (ROI). For analysis of spontaneous Ca^2+^-transients in astrocytes, three ROIs located at branches and one ROI located at the soma were chosen. Overall Ca^2+^ response to agonists of eCB agonist or synaptic stimulation was quantified using ROIs covering the whole cell image.

### (d) Measurement of extracellular concentration of ATP and d-serine in the brain tissue

The concentration of ATP within cortical slices was measured using microelectrode biosensors obtained from Sarissa Biomedical Ltd (Coventry, UK). A detailed description of the properties of biosensors and the recording procedure has been published previously [30]. Briefly, biosensors consisted of ATP or d-serine metabolizing enzymes immobilized within a matrix on thin (25–50 µM) Pt/Ir wire. This allowed insertion of the sensors into the cortical slice and minimized the influence of a layer of dead surface tissue. Concentrations of ATP and d-serine were measured simultaneously. The concentration of ATP or d-serine has been calculated from difference in the signals of two sensors: a screened ATP- or d-serine sensor and screened null-sensor, possessing the matrix but no enzymes. This allowed compensation for release of any non-specific electro-active interferents. Biosensors show a linear response to increasing concentration of ATP and d-serine and have a rise time of less than 10 s [30]. Biosensors were calibrated with known concentrations (10 μM) of ATP and d-serine before the slice was present in the perfusion chamber and after the slice had been removed. This allowed compensation of any reduction in sensitivity during the experiment. Biosensor signals were acquired at 1 kHz with a 1400 CED interface and analysed using spike 6.1 software (Cambridge Electronics Design, Cambridge, UK).

### (e) Data analysis

All data are presented as mean ± s.d.; the statistical significance of difference between data groups was tested by two-population *t*-test, unless indicated otherwise. The spontaneous transmembrane currents recorded in neurons were analysed off-line using methods described previously [25,27–29]. The amplitude distributions of spontaneous and evoked currents were analysed with the aid of probability density functions and likelihood maximization techniques [25,29]; all histograms shown were calculated as probability density functions. The amplitude distributions were fitted with either multi-quantal binomial model or bi-modal function consisting of two Gaussians with variable peak location, width and amplitude. The decay time distributions were fitted with bi-modal functions. Parameters of models were fit using likelihood maximization routine. To monitor and analyse the time course of changes in the amplitude and frequency of spontaneous currents, the amplitude and frequency were averaged over the 1 min time window.

## 3. Results

### (a) eCBs trigger Ca^2+^-signalling and release of ATP and d-serine from neocortical astrocytes *in situ*

To verify that astroglial CB1 receptors are able to induce significant elevation of intracellular Ca^2+^ level, we applied AEA to neocortical slices of GFAP-EGFP (GFEC) transgenic mice and dn-SNARE mice and monitored astroglial Ca^2+^-signalling using 2-photon fluorescent microscopy. Conveniently, a large proportion of neocortical astrocytes in these mice exhibits EGFP fluorescence, which helped to identify them. We also compared Ca^2+^ in astrocytes of dn-SNARE mice and in astrocytes of their WT littermates. Astrocytes were loaded with fluorescent dyes Rhod-2AM (GFEC and dn-SNARE mice) or OGB-2AM and sulforhodamine 101 (WT) and identified by astrocyte-specific fluorescence and electrophysiological properties as described in Material and methods.

**Figure 1.**
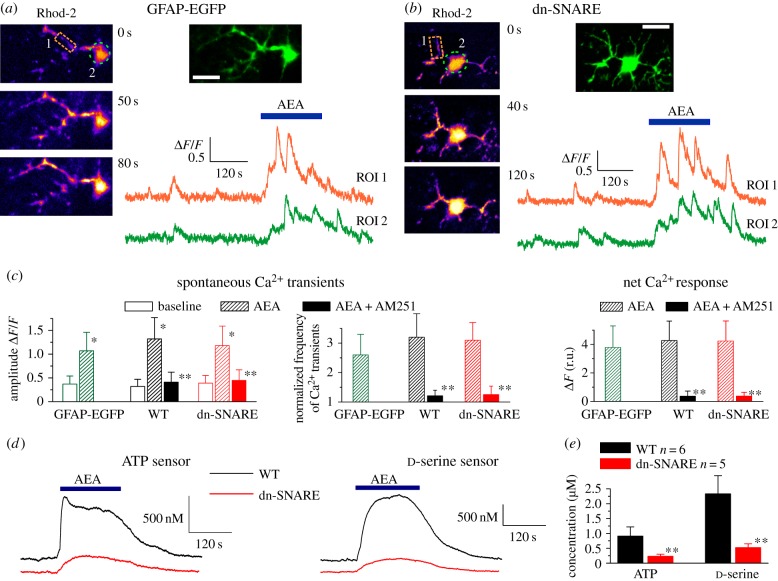
Cannabinoid receptors activate astrocytic Ca^2+^-signalling and release of gliotransmitters. (*a*–*c*) Ca^2+^-signalling activated by the bath application of 500 nM of anandamide (AEA) to neocortical slices. (*a*,*b*) Representative multi-photon images of EGFP fluorescence and pseudo-colour images of Rhod-2 fluorescence recorded in the astrocytes from GFAP-EGFP (*a*) and dn-SNARE (*b*) mice during AEA application at the times indicated. Scale bars, 10 μm. Graphs below show the time course of Rhod-2 fluorescence averaged over regions indicated in the fluorescence image. Note the marked spontaneous elevations in the Ca^2+^ level, which were enhanced by application of AEA. (*c*) The pooled data on peak amplitude and frequency of spontaneous Ca^2+^ transients and the net response to AEA recorded in astrocytes of different mouse strains in control and in the presence of CB1 receptor antagonist AM251 (1 μM). Data shown as mean ± s.d. for six cells in control (for each strain) and five cells under AM251 (WT and dn-SNARE). Frequency of spontaneous transients (middle graph) was measured within 3 min after application of AEA and was normalized to baseline value. Net response was evaluated as an integral Ca^2+^ signal measured during 3 min after AEA application, averaged over the whole cell image and normalized to the integral Ca^2+^ signal measured during 3 min before AEA application. Single asterisk (*) indicates statistical significance of effect of AEA on the peak amplitude of Ca^2+^ transients, and double asterisks (**) indicate significance of inhibitory effect of AM251; *p* < 0.01 given by *t*-test in both cases. (*d*,*e*) AEA-activated release of ATP and d-serine in the neocortex *in situ* was detected using microelectrode sensors. (*d*) The representative responses of cortical slices of WT and dn-SNARE mice to the application of 500 nM AEA were recorded using microelectrode sensors to ATP and d-serine placed in the layer II/III (see Material and methods). The data are shown as an elevation relative to the resting concentration. (*e*) The pooled data on the peak magnitude of ATP and d-serine transients evoked by application of AEA; data shown as mean ± s.d. for number of experiments indicated. Double asterisks (**) indicate statistical significance of difference in the magnitude of ATP and d-serine responses between WT and dn-SNARE mice, *p* < 0.01 (*t*-test). The significant reduction in the AEA-evoked responses in the cortical slices from dn-SNARE mice strongly supports the vesicular mechanism of ATP and d-serine release from astrocytes. (Online version in colour.)

In baseline conditions (before application of AEA), astrocytes of all mice strains exhibited spontaneous Ca^2+^ transients, which were more prominent in the branches (figure 1*a*,*b*). The average frequency of transients per astrocyte varied in the range of 0.5–2.1 min^−1^ for all mice strains. Bath application of 500 nM AEA induced robust Ca^2+^ elevation both in the soma and branches and increased the amplitude and frequency of spontaneous Ca^2+^ transients (figure 1*a*,*b*). There was no statistically significant difference in the action of AEA between astrocytes of different strains (figure 1*c*). It has to be emphasized that, at the concentration used, AEA selectively activates CB1 receptors. The specificity of AEA action was confirmed by inhibition with AM251, a selective antagonist of the CB1 receptor. Application of AM251 (1 μM) produced a moderate effect on spontaneous Ca^2+^ transients but significantly decreased the response to AEA in five WT and five dn-SNARE astrocytes (figure 1*c*). Although it was unlikely that application of AEA could induce release of neurotransmitters from nerve terminals activating a secondary response in astrocytes, we verified the lack of a significant neuronal component in the Ca^2+^ response by applying AEA in the presence of 1 μM tetrodotoxin (TTX). Application of TTX did not produce notable effect on AEA-induced Ca^2+^ response in four WT and five dn-SNARE astrocytes (data not shown). Combined, these results show that CB1 receptors can bring substantial contribution to Ca^2+^-signalling in the neocortical astrocytes. Importantly, our data show that expression of dn-SNARE protein in astrocytes did not affect eCB-mediated signalling.

To investigate the release of gliotransmitters that could plausibly follow the activation of CB1 receptors in astrocytes, we used microelectrode biosensors to monitor the concentration of ATP and d-serine in the neocortical tissue. This technique has been used previously for evaluation of transmitter release in several brain areas [25,30]. Activation of intracellular Ca^2+^ in astrocytes by 500 nM AEA induced a robust increase in the levels of extracellular ATP and d-serine in the cortical tissues of WT mice. The eCB-induced elevation in the ambient concentration of extracellular ATP and d-serine reached 0.9 ± 0.3 μM and 2.3 ± 0.8 μM, respectively (figure 1*d*,*e*). The eCB-induced elevation in concentrations of ATP and d-serine was decreased in the dn-SNARE-expressing mice by 74 ± 17% (*n* = 5) and 83 ± 15% (*n* = 5), respectively, as compared with WT littermates (figure 1*d*,*e*). This result strongly supports the astroglial origin and vesicular nature of ATP and d-serine release in response to AEA. It is worth noting that one could not expect a complete inhibition of astroglial exocytosis in the neocortex of dn-SNARE mice because some proportion of astrocytes did not express dn-SNARE [24,26].

### (b) eCBs trigger vesicular release of ATP from acutely isolated astrocytes

**Figure 2.**
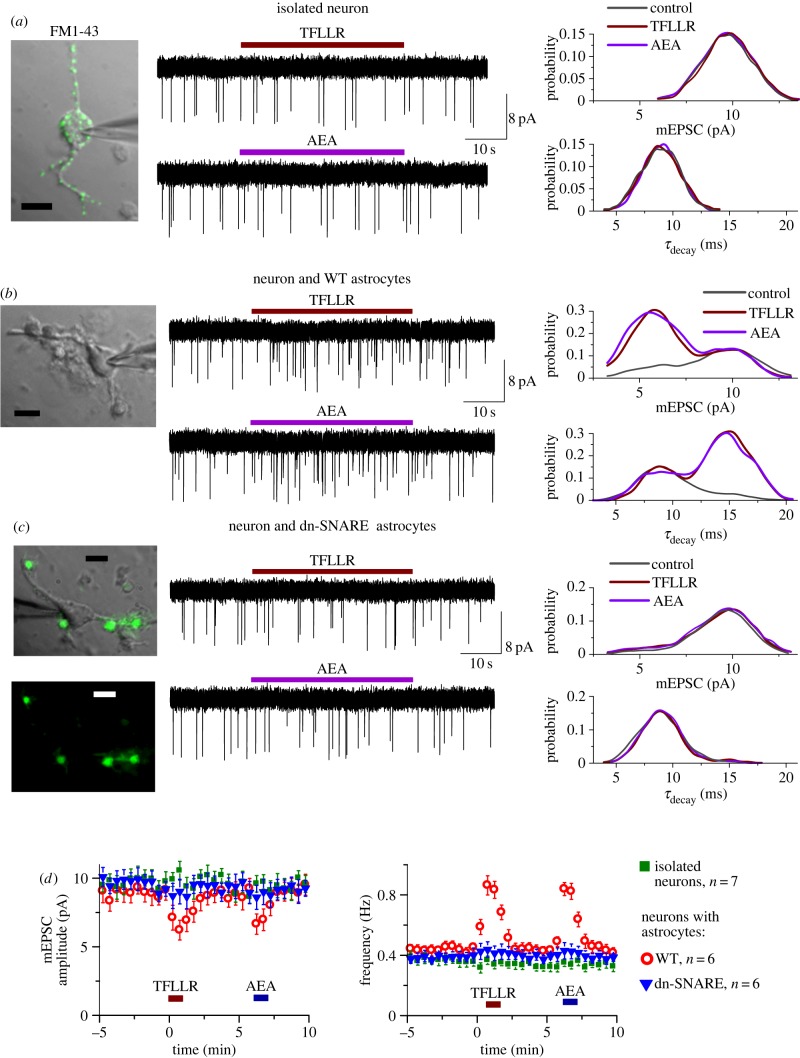
(*Overleaf*.) Detection of ATP release from astrocytes using modified ‘sniffer-cell’ method. Parameters of the technique of non-enzymatic cell dissociation [12,25,28,29] were optimized to yield either isolated neurons (*a*) or neurons attached to one or a few astrocytes (*b*,*c*). This dissociation technique allows neurons to retain functional synapses [12]. Whole-cell currents were recorded in the acutely dissociated neocortical pyramidal neurons at a membrane potential of −80 mV in the presence of CNQX (50 µM), D-APV (30 µM) and picrotoxin (100 µM). Currents recorded in neocortical neurons under these conditions are mediated predominantly by P2X receptors to ATP [25,29]. This can make neurons native detectors of ATP release. Astrocytes were activated by 1 min-long application of agonists of PAR-1 receptors (TFLLR) and CB1 receptors (AEA). (*a*) Whole-cell currents were recorded in the isolated neuron simultaneously with staining with synaptic vesicular marker FM1-43. Prior to recording, the cell was pre-incubated with 3 μM FM1-43 for 15 min, then dye was washed out for 15 min. From left to right: representative fluorescent and gradient contrast image of neuron showing punctate staining with FM1-43, the traces showing spontaneous synaptic currents and distributions of mEPSCs amplitude and decay time pooled for seven neurons. Note that application of TFLLR and AEA did not change the frequency of mEPSCs and did not alter their amplitude or decay time distribution. (*b*) Similar recordings were made in the neuron attached to a few astrocytes after their dissociation from neocortex of WT mice. After recordings, identification of astrocytes was confirmed by their functional properties as described in Material and methods. Both TFLLR and AEA caused the appearance of a large number of ATP-mediated mEPSCs. This was associated with appearance of additional peaks in the amplitude and decay time distributions, correspondingly at lower amplitude and slower decay time. (*c*) Both TFLLR and AEA did not have marked effect when neuron and astrocytes were dissociated from neocortex of dn-SNARE mice. Green fluorescence confirms the dn-SNARE expression in the astrocytes attached to the neuron. (*d*) Time course of changes in the amplitude and frequency of spontaneous purinergic currents during application of TFLLR and AEA (10 µM). Dots represent values averaged over a 30 s time window; data are shown as mean ± s.d. for number of neurons as indicated. Difference between WT and dn-SNARE mice in the effect of TFLLR and AEA on mEPSC frequency was significant with *p* < 0.005 (*t*-test). (Online version in colour.)

To verify that CB1 receptors trigger a vesicular release of gliotransmitters, in the next line of experiments we used a ‘sniffer’-cell approach to detect a release of ATP from acutely isolated astrocytes. In our previous work, we demonstrated that neocortical neurons express functional P2X receptors [25,29] and these receptors can be activated by ATP released from astrocytes [29]. Thus, acutely dissociated cortical neurons could serve as a native sensor for ATP. We used a technique of non-enzymatic vibro-dissociation which allows retention of functional membrane receptors at the cell surface [25,28,29], including P2X receptors. Another benefit of this technique is the ability to retain a fraction of functional synapses as well [12], which can be verified by staining with FM1-43 and the presence of miniature spontaneous synaptic currents (figure 2*a*). Furthermore, adjustment of the parameters of vibro-dissociation allowed us to dissociate neurons with a few astrocytes attached (figure 2*b*–*d*), thereby retaining a certain proportion of intimate contact between thin astrocytic processes and neuronal membrane. Such a neuron–astrocyte ‘bundle’ could work as a good model of the glia–neuron interaction unit, enabling efficient activation of astrocytes and direct monitoring of neuronal response. In comparison to bath application of extracellular agents to the whole slice, the ‘neuron–astrocyte bundle’ has the advantage of better control of drug application and a lack of side-effects of massive activation of the astrocyte network and poly-synaptic connections.

We recorded whole-cell currents in the acutely dissociated neocortical pyramidal neurons at a membrane potential of −80 mV in the presence of CNQX (50 µM), D-APV (30 µM) and picrotoxin (100 µM). Similarly to our previous experiments [25,29], we observed residual non-glutamatergic miniature spontaneous synaptic currents (figure 2). These non-glutamatergic excitatory spontaneous currents (mEPSCs) were completely abolished by application of specific P2X receptors antagonists PPADS (10 µM) and 5-BDBD (5 µM) in all five neurons tested (data not shown). Based on these data as well as our previous work [25,29], the pulsatile inward currents observed in cortical neurons in the presence of glutamatergic and GABAergic antagonists can be confidently attributed to the ATP receptors.

In order to activate CB1 receptor-mediated Ca^2+^-signalling in astrocytes, we applied 500 nM AEA. For a positive control, we used 5 µM TFLLR, an agonists of the PAR-1 receptor, which activate Ca^2+^ signalling selectively in astrocytes [25]. As a negative control, we used fully isolated neurons (figure 2*a*) that were devoid of any astrocytes. These neurons exhibited purinergic mEPSCs with average amplitude of 9.8 ± 2.2 pA and average decay time constant of 8.9 ± 1.7 ms (*n* = 7). Neither TFLLR nor AEA produced marked changes in the amplitude or frequency of spontaneous purinergic mEPSCs in all of the seven fully isolated neurons tested.

In contrast to fully isolated neurons, purinergic mEPSCs recorded in dissociated neurons that had WT astrocytes attached (figure 2*b*) exhibited bimodal amplitude distributions with peaks at 5.9 ± 1.4 pA and 9.9 ± 2.6 pA (*n* = 6). The distributions of mEPSC decay time in these neurons had peaks at 8.8 ± 1.1 ms and 15.1 ± 2.2 ms. Application of TFLLR caused a significant increase in the frequency of purinergic mEPSCs and decrease in their average amplitude (figure 2*b*,*d*) in all of six experiments. This effect was associated with selective increase in the number of events with smaller amplitude and slower decay kinetics (figure 2*b*, right column). Application of TFLLR did not cause marked increase in the number of purinergic currents in neurons attached to the dn-SNARE-expressing astrocytes (figure 2*c*,*d*). This observation closely agrees with our previous experiments carried out in the neocortical slices where we demonstrated that TFLLR-induced purinergic mEPSCs of smaller amplitude and slower kinetics originated from the vesicular release of ATP from astrocytes [25].

Similarly to TFLLR, application of AEA significantly increased the frequency of purinergic currents of slower kinetic and smaller amplitude (figure 2*b*,*d*) in the neurons connected to the WT but not dn-SNARE astrocytes. It has to be noted that neurons were perfused with extracellular solution containing 10 mM EGTA which should effectively inhibit putative retrograde release of neurotransmitters, as shown in Duguid *et al.* [12]. Thus, our data provide strong evidence that glial CB1 receptors are able to trigger vesicular release of gliotransmitters.

### (c) CB1 receptors contribute to control of astroglial exocytosis *in situ*

**Figure 3.**
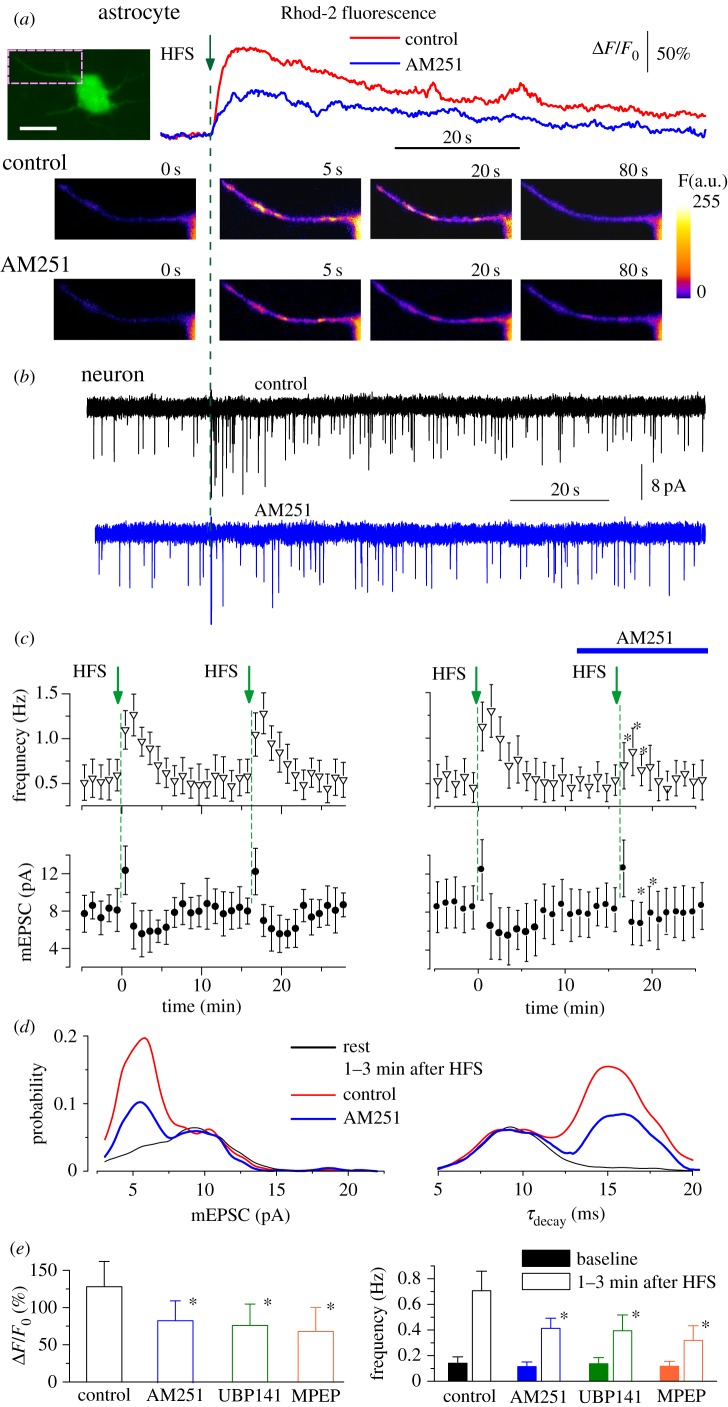
(*Overleaf*.) Attenuation of Ca^2+^-signalling in cortical astrocytes inhibits the release of ATP. Ca^2+^-signalling was monitored in cortical astrocytes loaded with Rhod-2AM using multi-photon fluorescent microscopy simultaneously with whole-cell recording of spontaneous transmembrane currents in the pyramidal neurons of the layer II/III of somatosensory cortex of WT mice. Spontaneous currents recorded in the pyramidal neurons at holding potential of −80 mV in presence of picrotoxin (100 µM), CNQX (50 µM) and D-AP5 (30 µM) were mediated by P2X receptors, as shown previously [25,29]. Recordings were made before and after application of endocannabinoid CB1 receptor antagonist AM251 (1 µM). (*a*) The single theta-burst of 100 Hz stimulation (HFS) was delivered at zero time to trigger Ca^2+^ elevation in the astrocyte. Graphs show the time course of Rhod-2 fluorescence averaged over a single astrocytic process indicated in the GFAP-EGFP fluorescence image of the astrocyte (scale bar 5 μm). The representative pseudo-colour multi-photon images, recorded at the times indicated, are show below. (*b*) Astrocytic Ca^2+^ elevation was followed by the burst of spontaneous purinergic currents that had a smaller quantal amplitude and slower decay, as shown previously [29], see also figure 2. Inhibition of CB1 receptors with AM251 caused the decrease in the Ca^2+^ elevation in the astrocyte and attenuation of burst of purinergic activity in the neuron. (*c*) Time course of changes in the amplitude and frequency of purinergic mEPSCs recorded in the neocortical neurons in control (left column) and in the presence of AM251 (right column) as shown above. Each dot shows the average amplitude and frequency of spontaneous currents recorded in a 1 min time window in the pyramidal neurons; data are presented as mean ± s.d. for seven neurons. An asterisk (*) indicates a significant difference from the control value (*p* < 0.05). The decrease in amplitude and significant increase in frequency of purinergic mEPSCs after HFS episodes was repeatedly observed in the control but was reduced after application of AM251. (*d*) The amplitude and decay time distributions of purinergic mEPSCs recorded before and during 1–3 min after HFS (pooled data for six neurons) show the presence of a distinct population of spontaneous currents of smaller amplitude and slower kinetics which were attributed to the dn-SNARE-dependent ATP release from astrocytes, as shown previously [25] and in figure 2. The increase in the fraction of slower mEPCSs was considerably reduced after application of AM251. (*e*) A similar experimental paradigm was used with application of antagonist of mGluR5 (MPEP, 10 μM) and subunit-specific NMDA receptor antagonist UBP141 (3 µM), which has been shown to selectively inhibit NMDA receptors in cortical astrocytes without affecting neuronal receptors [27]. These receptors have been shown to mediate Ca^2+^-signalling in astrocytes and have been suggested to trigger release of gliotransmitters [25,33]. Diagrams show the peak magnitude of HFS-evoked Ca^2+^ transients in the astrocytes and frequency of slow purinergic mEPCSs averaged within a 3 min time window before (baseline) and after HFS train. Data are shown as mean ± s.d. for the following number of experiments: seven for AM251, six for UBP141 and four for MPEP. Statistical significance of the difference from the control values is indicated by an asterisk (*p < 0.05). All antagonists significantly decreased the astrocytic Ca^2+^ elevation and the frequency of slow glia-driven purinergic mEPSCs, indicating that release of ATP from cortical astrocytes *in situ* could be controlled synergistically by CB1, NMDA and mGluR5 receptors. (Online version in colour.)

The above data were obtained upon activation of astrocyte CB1 receptors by exogenous agonist, and it would be natural to ask whether endogenous eCBs could activate exocytosis from cortical astrocytes. We demonstrated previously that a short burst of HFS of intracortical afferents was able to elevate cytosolic Ca^2+^ level in the cortical astrocytes and trigger vesicular release of ATP. To explore a plausible involvement of CB1 receptors in this effect, we monitored purinergic spontaneous currents in the pyramidal neurons of neocortical slices at membrane potential of −80 mV in the presence of CNQX and picrotoxin (figure 3). Currents recorded in the neocortical neurons under these conditions were shown to be mediated solely by P2X receptors [25,29].

The short HFS train caused significant elevation of the frequency of purinergic mEPSCs in the pyramidal neurons of WT mice, this elevation being associated with increased number of mEPSCs of smaller amplitude and slower kinetics. We have shown previously that such mEPSCs originate from dn-SNARE-dependent release of ATP from astrocytes [25]. Thus, similarly to our present data obtained in the dissociated neurons (figure 2), appearance of slower purinergic mEPSCs can be used as a good read-out for glial exocytosis. We found that inhibition of CB1 receptors with AM251 considerably reduced the Ca^2+^ elevation triggered in the astrocytes by HFS train (figure 3*a*) and decreased the following burst of slow purinergic mEPSCs (figure 3*b*–*d*). This result indicates that eCB-mediated signals from neurons can activate exocytosis from astrocytes in physiological conditions. To estimate the putative impact of eCBs on glial exocytosis, we compared the modulatory effect produced by AM251 to the effect produced by antagonists of mGluR5 and NMDA receptors, reported to contribute to astrocytic Ca^2+^ signalling [27,28,31]. The effect of eCB antagonist was comparable to that of MPEP, an antagonist of mGluR5 [31], and UBP141, a selective antagonist of glial NMDA receptors [27,28]. This result suggests that eCB receptors can bring notable contribution to the Ca^2+^-dependent modulation of exocytosis (at least, of ATP) in the neocortical astrocytes (figure 3*e*).

### (d) Astroglial CB1 receptors modulate long-term plasticity in neocortex

Our experiments showed that CB1 receptors can activate release of ATP and d-serine from cortical astrocytes (figure 1). The release of ATP and d-serine from astrocytes has been shown previously to regulate synaptic plasticity in the hippocampus [23,24]. The role of these gliotransmitters in the modulation of synaptic plasticity in the neocortex remains almost unexplored. We have shown previously that astrocyte-derived ATP can downregulate phasic and tonic GABAergic transmission in the neocortical neurons [25]. Regulation of neural excitability by phasic and tonic GABA conductance can regulate the long-term synaptic plasticity in the neocortex and hippocampus [32,33]. Thus, one could expect that eCB-mediated release of ATP might affect the induction of LTP in the neocortical neurons.

**Figure 4.**
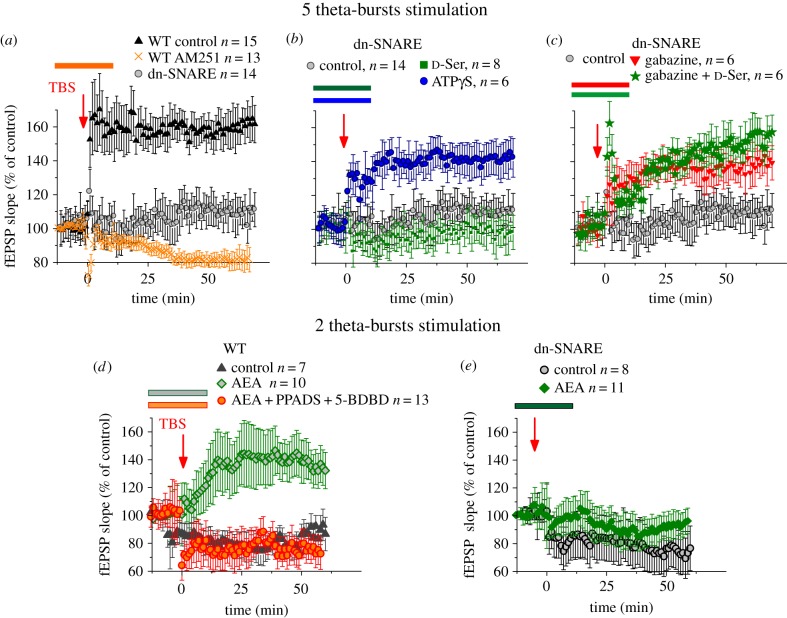
Interplay between gliotransmitters modulates synaptic plasticity in the neocortex. Long-term potentiation of field EPSPs in the layer II/III of somatosensory cortex was induced by five (*a*–*c*) or two (*d*,*e*) episodes of TBS delivered at zero time. Dots in the graphs represent the average of six consecutive EPSPs; data are shown as mean ± s.d. for number of experiments indicated. Data were normalized to the EPSP slope averaged over a 10 min period prior to the TBS. All drugs were applied 15 min prior induction of LTP and were washed out 10 min after TBS present during the course of experiment, as indicated in the graphs by corresponding colour bars. (*a*) In WT mice, five episodes of TBS induce robust LTP in the control but not after inhibition of CB1 receptors with AM251. Impairment of exocytosis of gliotransmitters dramatically reduced the magnitude of LTP in the dn-SNARE mice. (*b*) Application of exogenous d-serine (10 µM) did not rescue induction of LTP in the dn-SNARE mice, whereas application of selective agonist of P2X purinoreceptors ATPγS (10 µM) rescued the LTP. This result suggests that downregulation of inhibitory signalling by ATP released from astrocytes [25] can modulate the synaptic plasticity in neocortex. (*c*) Feasibility of this mechanism is supported by significant increase in the LTP magnitude in dn-SNARE mice caused by GABA receptor antagonist gabazine (150 nM) on LTP. Under action of gabazine, d-serine became capable of increasing the LTP magnitude. (*d*) The weaker 2-TBS stimulation did not induce the LTP in WT mice in control but application of AEA (500 nM) enabled the induction of LTP. This effect was prevented by inhibition of P2X receptors with selective antagonists PPADS and 5-BDBD. (*e*) Application of AEA did not facilitate LTP induction in the dn-SNARE mice, suggesting the importance of glial exocytosis for the action of AEA. Combined, these results suggest the importance of eCB-mediated astroglial signalling and regulation of neuronal excitability by purinoreceptors activated by astrocyte-driven ATP. (Online version in colour.)

We investigated the long-term potentiation of the field excitatory post-synaptic potentials (EPSPs) in layer II/III of the somatosensory cortex of WT and dn-SNARE mice. The EPSPs were evoked by the stimulation of the same neuronal afferents descending from layers IV–V as in the experiments described above (figure 3). Potentiation of EPSPs was induced by a few episodes of TBS (see Material and methods). In WT mice, five episodes of TBS (5 TBS) induced robust LTP in all 15 trials (figure 4*a*). The extent of LTP of cortical EPSPs was dramatically reduced in the dn-SNARE mice (figure 4*a*), indicating the crucial importance of glial exocytosis. Application of the CB1 receptor antagonist AM251 also inhibited the induction of LTP, suggesting the importance of eCB-mediated signalling. To elucidate putative roles for glia-derived ATP and d-serine, we tried to rescue the LTP in dn-SNARE mice by extracellular application of these compounds. Surprisingly, application of extracellular d-serine did not rescue the induction of LTP in the neocortex (figure 4*b*). The induction of LTP in the dn-SNARE mice was, however, rescued by application of the non-hydrolysable selective agonist of P2X receptors ATPγS (figure 4*b*).

These results indicate the importance of vesicular release of ATP from astrocytes for synaptic plasticity in the neocortex. One of the putative mechanisms of ATP action could be lowering the threshold of LTP induction via downregulation of neuronal GABAA receptors caused by activation of post-synaptic P2X receptors. The stronger GABA-mediated inhibition in the dn-SNARE mice [25] and, consequently, insufficient depolarization of post-synaptic neurons for activation of NMDA receptors may explain the lack of positive action of d-serine during the LTP induction in the neocortex. The feasibility of this mechanism was corroborated by our finding that attenuation of GABA-mediated inhibition by gabazine (150 nM) facilitated the induction of LTP in the dn-SNARE mice (figure 4*c*). Moreover, application of d-serine in the presence of gabazine increased the LTP of fEPSPs in the neocortex (figure 4*c*), implying that attenuation of GABAergic inhibitory transmission can facilitate activation of NMDA receptors and thus lies upstream of positive modulation of NMDA receptors by d-serine.

The importance of glial CB1 receptors for neocortical plasticity was corroborated by experiments on induction of LTP by a weaker stimulus. In control conditions, two theta-bursts of HFS did not induce LTP (figure 4*d*) in any of seven trials. The threshold for LTP induction was three theta-bursts in our experimental conditions (data not shown). When the sub-threshold stimulation was applied in the presence of AEA, it became able to induce LTP. The effect of AEA was abolished by selective inhibition of P2X receptors with 10 μM PPADS and 5 μM 5-BDBD (figure 4*d*). This result strongly suggests that CB1 receptor-mediated facilitation of LTP relies on ATP release and activity of ATP receptors. Importantly, application of AEA did not facilitate LTP induction in the dn-SNARE mice (figure 4*e*). Combined, these results strongly suggest that CB1 receptors contribute to regulation of exocytosis of gliotransmitters, in particular ATP, and this pathway is important for synaptic plasticity in the neocortex.

## 4. Discussion

Our data on the substantial contribution of CB1 receptors to Ca^2+^-signalling in neocortical astrocytes closely agree with previous observations made in the hippocampus and neocortex [10,11]. We also have shown that CB1 receptors can activate SNARE-dependent release of ATP and d-serine. Moreover, we have found that eCB-activated release of ATP from astrocytes can directly activate post-synaptic P2X receptors in the neocortical neurons (figures 2 and 3). So, we showed for the first time a presence of clear purinergic component of eCB-mediated glia–neuron communication.

Traditionally, the action of glia-driven ATP on neuronal signalling was considered to be mediated mainly by pre-synaptic adenosine receptors activated after breakdown of ATP to adenosine [22,24,26]. We have found recently that glia-derived ATP can activate post-synaptic P2X receptors in the adjacent neurons and cause a downregulation of GABA receptors by a post-synaptic mechanism [25]. Our present results indicate that this pathway can be involved in modulation of synaptic plasticity in neocortex. Our data also suggest that glia-driven ATP can modulate neuronal signalling, acting upstream of its counterpart gliotransmitters d-serine and glutamate. Interplay between purinergic and glutamatergic components of eCB-activated gliotransmission might explain the diversity of effects of CB1 receptors on synaptic transmission in the neocortex and hippocampus.

To conclude, our results strongly support the physiological importance of astroglial cannabinoid signalling and exocytosis of gliotransmitters for communication between astrocytes and neurons and modulation of synaptic plasticity.

## Notes

All animal work has been carried out in accordance with UK legislation and the ‘3R’ strategy; research has not involved non-human primates.
